# Evidence for facultative migratory flight behavior in *Helicoverpa armigera* (Noctuidae: Lepidoptera) in India

**DOI:** 10.1371/journal.pone.0245665

**Published:** 2021-01-22

**Authors:** Patil Jyothi, Prabhuraj Aralimarad, Vijaya Wali, Shivansh Dave, M. Bheemanna, J. Ashoka, Patil Shivayogiyappa, Ka S. Lim, Jason W. Chapman, Sanjay P. Sane

**Affiliations:** 1 Department of Agricultural Entomology, University of Agricultural Sciences, Raichur, Karnataka, India; 2 Department of Agricultural Statistics, University of Agricultural Sciences, Raichur, Karnataka, India; 3 National Centre for Biological Sciences, Tata Institute of Fundamental Research, GKVK campus, Bengaluru, India; 4 Department of Agro-Ecology, Rothamsted Research, Harpenden, Hertfordshire, United Kingdom; 5 Centre of Ecology and Conservation, University of Exeter, Penryn, Cornwall, United Kingdom; 6 Department of Entomology, College of Plant Protection, Nanjing Agricultural University, Nanjing, People’s Republic of China; USDA Agricultural Research Service, UNITED STATES

## Abstract

Despite its deleterious impact on farming and agriculture, the physiology and energetics of insect migration is poorly understood due to our inability to track their individual movements in the field. Many insects, e.g. monarch butterflies, *Danaus plexippus* (L.), are facultative migrants. Hence, it is important to establish whether specific insect populations in particular areas migrate. The polyphagous insect, *Helicoverpa armigera* (Hübner), is especially interesting in this regard due to its impact on a variety of crops. Here, we used a laboratory-based flight mill assay to show that *Helicoverpa armigera* populations clearly demonstrate facultative migration in South India. Based on various flight parameters, we categorized male and female moths as long, medium or short distance fliers. A significant proportion of moths exhibited long-distance flight behavior covering more than 10 km in a single night, averaging about 8 flight hours constituting 61% flight time in the test period. The maximum and average flight speeds of these long fliers were greater than in the other categories. Flight activity across sexes also varied; male moths exhibited better performance than female moths. Wing morphometric parameters including forewing length, wing loading, and wing aspect ratio were key in influencing long-distance flight. Whereas forewing length positively correlated with flight distance and duration, wing loading was negatively correlated.

## Introduction

Migratory insects undertake journeys ranging from a few meters to thousands of kilometers over land and water [1]. In a wide range of insects, such as dragonflies [2, 3], grasshoppers [4], beetles, butterflies and moths [5–7], seasonal movements and travel distance during migration varies with species, and often involves large numbers of individuals [8]. Vast numbers of noctuid moths regularly migrate between their summer and winter ranges separated by thousands of kilometers, undertaking nocturnal flights at altitudes of hundreds or thousands of meters [9–12]. These migrations are either obligate (i.e. independent of environmental factors and in habitats that support a single generation; [13]), or facultative (i.e. mainly depending on environmental cues experienced during development; [14, 15]). Because most insects are too small to be individually tracked during migration, knowledge about insect migration lags behind that of vertebrates. Nevertheless, insects are readily amenable to experimental manipulation and their migration can be studied using laboratory-based assays.

The study of migration and mass movement is especially important for insects that feed extensively on crops. Of insects that threaten crops, the moth *Helicoverpa armigera* (Hübner) has emerged in recent years as a key crop-pest in the old world. *Helicoverpa armigera* is a polyphagous pest with more than 300 host plants, ravaging several crops of the arid and semiarid tropics across the globe [16–18]. In India, it is a major pest of pigeon pea, chickpea, sunflower, sorghum, maize and tomato [19, 20]. The potential for extensive adult movements contributes greatly to the success of heliothines (includes *Helicoverpa*, *Heliothis*, *Chloridea*) as pests [16, 21, 22]. In southern India, *H*. *armigera* breeds throughout the year completing over eight generations/year [23]. During harsh summers, these moths adopt one of three survival strategies. Some moths undergo diapause [24] whereas others survive on non-seasonal crops [25]. The remaining population is thought to migrate to Central and Northern India to exploit the available resources. Under certain conditions *Helicoverpa* moths undertake long flights from one crop-growing area to another [21, 22, 26, 27]. Given their agricultural impact, it is essential to establish if *H*. *armigera* populations in India are indeed migratory. Several techniques, including mark-release-recapture [28], visual observations and radar technology have been used to document long-distance migration of *Helicoverpa* in Southern India [29]. However, due to their small size and nocturnal behaviour, it is difficult to track that the trajectories of individual moths over long distances [30]. Moreover, for facultative migrants it is necessary to investigate both long-distance migratory behavior, and the proportion of the population that migrates. Additionally, can morphological flight-related characters allow us to separate migrants from non-migrants?

To study flight physiology and behaviour, researchers have used laboratory-based flight-mill techniques in tethered insects, including *Helicoverpa* [31–33]. In general, tethered flight assays on insects provide insights that are elusive in larger animals. Such assays have proved useful in assessing flight behaviour of insects ranging from flies and true bugs to butterflies, moths, and beetles [33]. In some noctuids [13, 34–39] including *H*. *armigera*, migratory flight typically occurs early in adult life in the pre-reproductive period [40]. A better understanding of migratory flight would enable tracking population movements, estimating proportion of gene flow across migrating pathways, periods of emigration and immigration and designing better management strategies. Hence, we investigated the migratory behavior of the South Indian population of *H*. *armigera* using the tethered flight mill system.

## Material and methods

### Maintaining the adult population for flight study

To have a continuous moth population, culture in the form of fully grown larvae (preferably sixth instar) were collected from different cropping ecosystems such as pigeonpea, okra, pearl millet, castor, chickpea, and sunflower spread around 100 km of the study location (16.2043° N, 77.3345° E) from June 2016 to February 2017. Such collected larvae were weighed to select uniform sized caterpillars and reared individually in plastic vials (50 ml capacity) in an environmental chamber set at 27°C ± 1°C with 80 percent relative humidity and L:D of 12:12 h. During rearing, larvae were fed with the same host crop on which they were collected till pupation. Pupae thus formed were sexed, weighed and retained in the same vial for eclosion. The emerged adult moths were fed with 10 percent honey solution and weighed before tethering to flight mill. After the flight mill experiment, moths were killed to record various wing morphometrics.

### Tethered flight mill assay

**Fig 1:**
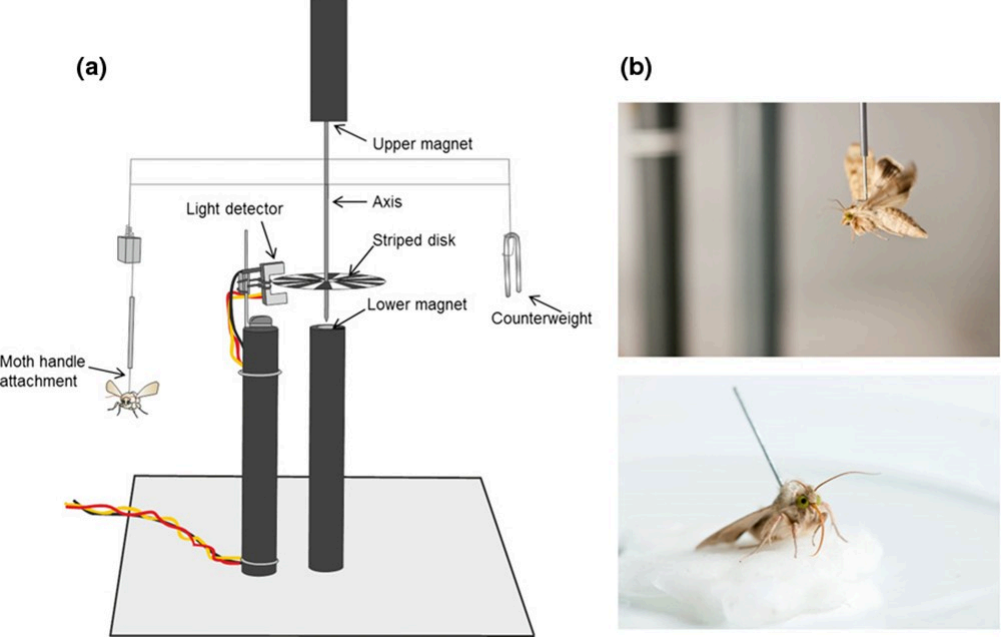
An example of a tethered flight mill for studying migratory flight under controlled conditions. (a) Schematic diagram of an individual rotational flight mill, showing the low-friction magnetic suspension which enables comparatively small insects to engage in sustained flight. (b) Experimental moth, *Helicoverpa armigera* attached with short handle to the dorsal surface of the thorax few hours before nocturnal flight. Figure and photos courtesy of Rothamsted Research Visual Communications Unit.

The flight-mill system was designed and developed by K.S.L. (Patent: [41]), and has 8 channels (arms) allowing 8 individual moths to be flown simultaneously. Each mill consists of a lightweight arm suspended between two magnets (Fig 1). The magnetic suspension provides an axis with minimal resistance, allowing even relatively weak fliers to turn the mill. The moth was attached to one end of the flight-mill arm and flew in a circular trajectory with a circumference of 50 cm. A small banded patterned disk attached to the axis turned with the arm, while a light-detector measured the number of turns to measure the distance flown and flight speed. The flight-mill is interfaced with a computer to log the flight data. An embedded microcontroller board recorded the distance flown by the insect to the nearest 10 cm and updated at five-second intervals. A similar flight-mill system was previously used to study moth flight ability [27, 32].

One-day old (<24 hours post-eclosion) moths were collected and cold-anesthetized in a freezer (-20°C) for 4–5 minutes to immobilize and to protect them from physical abrasion during tethering. The cold-anesthetized moth was placed on a plastic tethering platform held at 45°, with a central chamber in which the moth was held in place using perforated plastic net. The meso- and meta-thoracic terga were descaled with a fine hairbrush, and a metal tethering pin (3–4 cm height, 0.07 g weight) bent in a circular loop was glued to the descaled tergal plate with Cyanoacrylate superglue (evo bond). Each tethered moth was attached to an individual flight-mill channel. The flight mill containing eight such tethered moths (either males or females) was oused in a controlled environment chamber and flight data was recorded from 17.00 to 07.00 (14 hours). Dead or inactive moths during observation period were excluded from the analyses. In total 106 moths (52 males and 54 females) were subjected to flight behaviour study.

Flight performance in terms of total distance (TD), time spent in flying (TSF), per cent flight time (PFT), average (AFS) and maximum flight speed (MFS) of individual moths was plotted to observe the trend in the flight behaviour. Based on the flight performance 106 moths observed were classified as ‘short fliers’ (flight range of 0–5 km), ‘medium fliers’ (5–10 km), and ‘long fliers’ (>10 km) [27]. The above flight parameters viz., TD, TSF, AFS, MFS and PFT of individuals in the short, medium and long flier categories were analyzed with one-way analysis of variance (ANOVA) and the means (expressed along with SE) were separated using Tukey’s honestly significance difference test (HSD) using SPSS.16. We used the student t-test to compare how each parameter varied between sexes for each parameter, setting the significance level at P < 0.01.

Post-recording, the fore- and hindwing on one side (right) were detached using micro-scissors, and total wing length, width and area of each wing measured using a stereo-zoom binocular microscope (Nikon: SMZ 25) equipped with measurement software (NIS Elements F 4.00.00). We calculated wing aspect ratio, wing loading, and front wing quotient using standard formulae [42].

We processed flight data of individual moths for a 14-hour flight period (from beginning of dusk to end of dawn) using MATLAB [43]. Flight data were processed in R [44] to extract 16 flight variables (S1 Table in S1 File), of which 11 most informative variables were isolated using Principal Components Analysis (PCA) (S2 Table in S1 File). Using Pearson correlation and stepwise regression analysis (S3 and S4 Tables in S1 File), we established the relationship between flight variables (extracted from PCA) and adult morphometric variables (S5 Table in S1 File). Linear regression analyses were performed to test the effect of three most important wing morphometrics viz., forewing length, wing aspect ratio and wing loading with flight variables.

## Results

**Fig 2:**
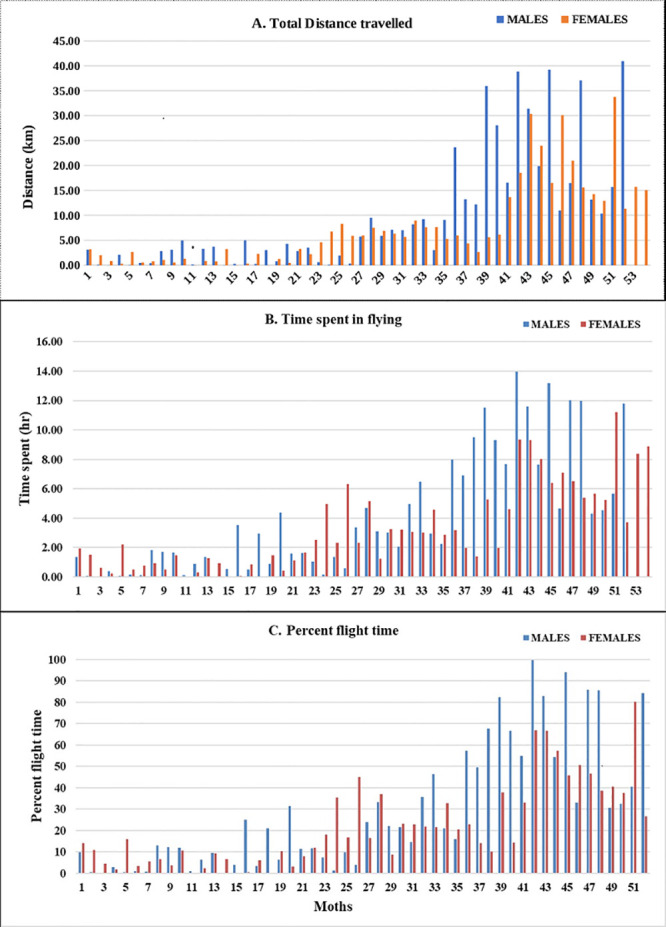
Graph representing flight variables exhibited by male and female moths of *H*. *armigera*. A) Total distance travelled B) Time spent in flying and C) Percent flight time.

**Fig 3:**
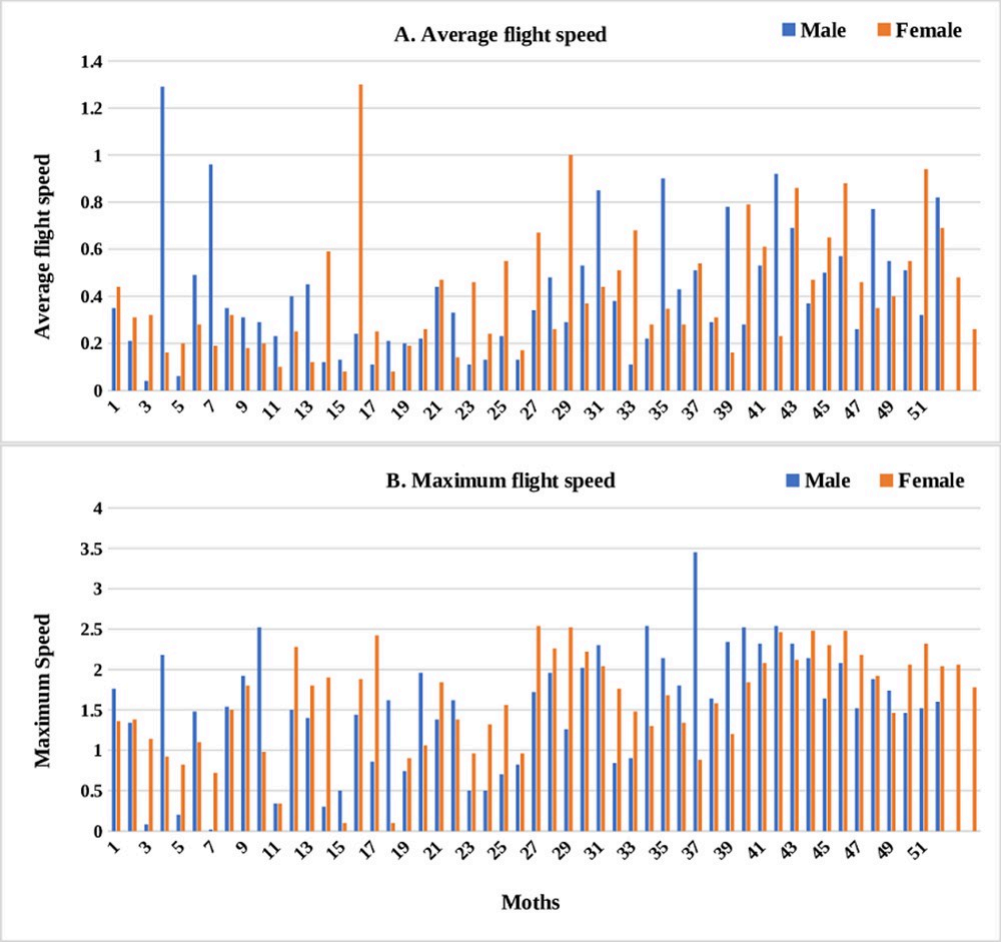
Graph representing flight variables exhibited by male and female moths of *H*. *armigera*. A) Average flight speed and B) Maximum flight speed.

Irrespective of sex, adult moths exhibited three distinct categories of flight behaviour with respect to total distance, time spent in flying and per cent flight time (Fig 2A, 2B and 2C). Of 106 moths, 49 exhibited short-range (0–5 km), 29 medium-range (5–10 km), and 31 long-range (> 10 km) flight. However, not much difference was observed with respect to average and maximum flight speed among the moths tested (Fig 3A and 3B).

Regardless of sex, moths of each category differed significantly with respect to time spent in flying, total distance, average and maximum flight speed, and percent flight duration. Mean time spent in flying were 8.19 h (± 0.52 h), 3.42 h (± 0.28 h), and 1.02 h (± 0.14 h) for long, medium and short fliers, respectively (F_c_ (2, 104) = 145.57, P < 0.01). Long-range fliers flew a distance of 21.81 ± 1.76 km, as compared to medium-range (6.62 ± 0.34 km) and short-range fliers (1.61 ± 0.22 km) (F_c_ (2, 104) = 132.99, P < 0.01). Long-range fliers attained a maximum speed of 2.07 m/s (± 0.08 m/s), not significantly greater than medium-range fliers (1.70 ± 0.10 m/s) but significantly faster than short-range fliers (1.18 ± 0.09 m/s) (F_c_ (2, 104) = 24.03, P < 0.01). Similarly, average flight speed was highest in long-range fliers (0.55 ± 0.04 m/s) followed by medium-range (0.45 ± 0.05 m/s) and short-range (0.30 ± 0.04 m/s) flying moths (F_c_ (2, 104) = 9.82, P < 0.01) (Table 1). Trends across the three categories remained unchanged when male and female moths were analyzed separately (Table 2). Time spent in flying by female long-range fliers exceeded medium-range and short-range fliers (F_c_ (2, 51) = 80.93, P < 0.01). Maximum (F_c_ (0.05, 2, 51) = 12.54, P < 0.01) and average flight speed (F_c_ (2, 51) = 5.26, P < 0.01) of long-range female fliers were on par with medium-range, but significantly greater than short-range females. Likewise, percent flight duration of long-range fliers was greater than medium- and short-range fliers, but on par with each other (F_c_ (2, 51) = 3.07, P < 0.05) (Table 2).

**Table 1:** Flight performance in short, medium and long range fliers of *H*. *armigera* (both female and male moths).

Flight category	No. of moths tested	Time spent in flying (hr)			Total distance (km)			Maximum flight speed (m/s)			Average flight speed (m/s)			Percent flight time		
		Min	Max	Mean ± SE	Min	Max	Mean ± SE	Min	Max	Mean ± SE	Min	Max	Mean ± SE	Min	Max	Mean ± SE
**Short range fliers (0 to 5km)**	49	0	4.38	1.02**±**0.14^a^	0	4.99	1.61**±**0.22^a^	0.02	2.52	1.18 **±**0.09^a^	0.04	1.3	0.30 **±** 0.04^a^	0.81	100	31.75**±**4.56^a^
**Medium range fliers (5 to 10 km)**	26	1.22	6.48	3.42**±**0.28^b^	2.61	9.50	6.62**±**0.34^b^	0.84	2.54	1.70 **±**0.10^b^	0.11	1	0.45 **±** 0.05^ab^	10.03	74.06	33.13**±**3.45^a^
**Long range fliers (> 10 km)**	31	3.72	13.96	8.19**±**0.52^c^	10.36	40.92	21.81**±**1.76^c^	1.46	3.45	2.07 **±** 0.08^b^	0.23	0.94	0.55 **±** 0.04^b^	27.46	99.85	61.36**±**3.71^b^
Total	**106**	**3.71±0.35**			**8.75± 0.99**			**1.57 ± 0.07**			**0.41±0.03**			**40.75±2.82**		
**F-value**		145.57**			132.99**			24.03**			9.82**			13.81**		
**p-value**		**0.000**			**0.000**			**0.000**			**0.000**			**0.000**		

SE-Standard error,

***p<0.0001,

**p<0.01,

*p<0.05.

The data of flight parameters are presented as mean ± SE.

Means in the same column followed by different letters are significantly different by Tukey’s HSD (P = 0.01).

**Table 2:** Flight performance in short, medium and long-range fliers of male and female *H*. *armigera* moths.

Flight category		No. of moths tested	Time spent in flying (hr)			Total distance (km)			Maximum flight speed (m/s)			Average flight speed (m/s)			Percent flight time		
			Min	Max	Mean ± SE	Min	Max	Mean ± SE	Min	Max	Mean ± SE	Min	Max	Mean ± SE	Min	Max	Mean ± SE
**Short range fliers (0 to 5km)**	**♀**	23	0.00	2.51	0.93±0.15^a^	0.00	4.60	1.40±0.27^a^	0.10	2.42	1.25±0.13^a^	0.08	1.30	0.30±0.05^a^	2.39	100.00	32.51**±**7.81^a^
	**♂**	26	0.01	4.38	1.10**±**0.22^a^	0.00	4.99	1.80**±**0.34^a^	0.02	2.52	1.12**±**0.14^a^	0.04	1.29	0.31**±**0.05^a^	0.81	100.00	31.07**±**5.27^a^
**Medium range fliers (5 to 10 km)**	**♀**	17	1.22	6.31	3.29±0.35^b^	2.61	8.97	6.31±0.37^b^	0.88	2.54	1.68±0.12^ab^	0.16	1.00	0.45±0.06^ab^	10.03	72.57	31.04**±**4.05^a^
	**♂**	9	2.04	6.48	3.64**±**0.48^b^	3.02	9.50	7.19**±**0.70^a^	0.84	2.54	1.74**±**0.20^ab^	0.11	0.90	0.46**±**0.09^b^	16.11	74.06	37.10**±**6.52^a^
**Long range fliers (> 10 km)**	**♀**	14	3.72	11.22	7.13±0.057^c^	11.34	33.76	19.48±1.94^c^	1.46	2.48	2.12±0.08^b^	0.23	0.94	0.56±0.06^b^	27.46	84.74	52.98**±**4.22^b^
	**♂**	17	4.28	13.96	9.07**±**0.77^c^	10.36	40.92	23.74**±**2.74^b^	1.46	3.45	2.03**±**0.13^b^	0.26	0.92	0.54**±**0.05^b^	32.89	99.85	68.26**±**5.36^b^
**Total**	**♀**	54	3.28±0.39			7.63±1.13			1.61±0.08			0.41±0.04			37.35±3.89		
	**♂**	52	4.15±0.57			9.90±1.65			1.53±0.10			0.41±0.04			44.27±4.06		
**F-value**	**♀**	80.93**				97.05**			12.54**			5.26**			3.07*		
	**♂**	75.99**				57.11**			11.29**			4.35*			12.51**		
**p-value**	**♀**	**0.000**				**0.000**			**0.011**			**0.000**			**0.052**		
	**♂**	**0.000**				**0.000**			**0.000**			**0.023**			**0.000**		

SE-Standard error,

***p<0.0001,

**p<0.01,

*p<0.05

The data of flight parameters are presented as mean ± SE.

Means in the same column followed by different letters are significantly different by Tukey’s HSD (P = 0.01).

Greater flight duration in males was recorded in long-range fliers followed by medium- and short-range fliers (F_c_ (2, 49) = 75.99, P < 0.01). Maximum flight speed in long-range fliers was similar to medium-range fliers but significantly exceeded short-range fliers (F_c_ (2, 49) = 11.29, P < 0.01). Average flight speed (F_c_ (2, 49) = 4.35, P < 0.02) and percent flight time (F_c_ (2, 49) = 12.51, P < 0.01) (Table 2) followed similar trends.

**Fig 4:**
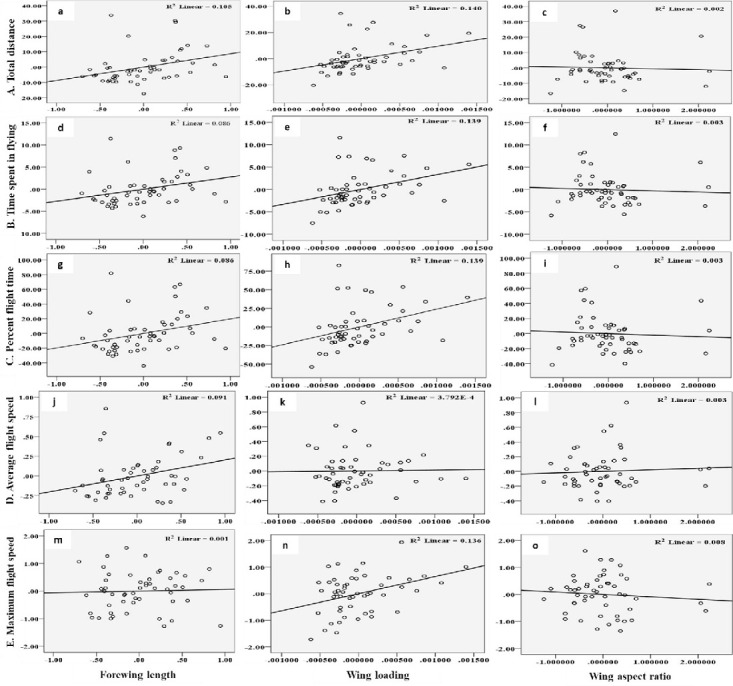
Graphs showing relationship between flight variables (dependent) with wing morphometrics (independent) in male moths of *H*. *armigera*. A) Total distance (a-c) B) Time spent in flying (d-f) C) Percent flight time (g-i) D) Average flight speed (j-l) and E) Maximum flight speed (m-o) as influenced by Forewing length, Wing loading and Wing aspect ratio.

The flight behaviour in adult moths with respect to total distance travelled, time spent in flying and percent flight time appears to be greatly influenced by three important wing morphometrics viz., forewing length (FWL), wing loading (WL) and wing aspect ratio (WAR). In male moths, total distance travelled exhibited a strong positive relation with FWL and WL but negative relation with WAR (Eq (1a); Fig 4a–4c). Correspondingly, time spent in flying by adult moths recorded significant positive relation with FWL and WL but nonsignificant negative relation with WAR (Eq (1b); Fig 4d–4f). The percent flight time which is the resultant of TSF recorded significant positive relation with FWL, positive but nonsignificant relation with WL and nonsignificant negative relation with WAR (Eq (1c); Fig 4g–4i). However, average flight speed though appears to be positively influenced by FWL, WL and WAR but is nonsignificant (Eq (1d); Fig 4j–4l). Similarly, maximum flight speed also recorded positive but nonsignificant relation with FWL and WL but negative relation with WAR (Eq (1e); Fig 4m–4o).

$${\text{Y}}_{\text{TD}}=\;-116.924+8.719\;*{\text{X}}_{1\;}+9521.283**{\text{X}}_{2}-0.577{\text{X}}_{3}+\varepsilon_{0}$$ (1a)

$${\text{Y}}_{\text{TSF}}=\;-36.117+2.750*{\text{X}}_{1\;}+3345.211**{\text{X}}_{2}-0.278{\text{X}}_{3}+\varepsilon_{0}$$ (1b)

$${\text{Y}}_{\text{PFT}}=-257.976+19.645*{\text{X}}_{1}+23894.361{\text{X}}_{2}-1.983{\text{X}}_{3}+\varepsilon_{0}$$ (1c)

$${\text{Y}}_{\text{AFS}}=\;-2.070+0.203*{\text{X}}_{1}+11.584{\text{X}}_{2}+0.20{\text{X}}_{3}+\varepsilon_{0}$$ (1d)

$${\text{Y}}_{\text{MFS}}=\;-0.727+0.058{\text{X}}_{1}+645.40**6{\text{X}}_{2}-0.090{\text{X}}_{3}+\varepsilon_{0}$$ (1e)

Where,

X_1_- Forewing length

X_2_- Wing loading

X_3_- Wing aspect ratio

**Fig 5:**
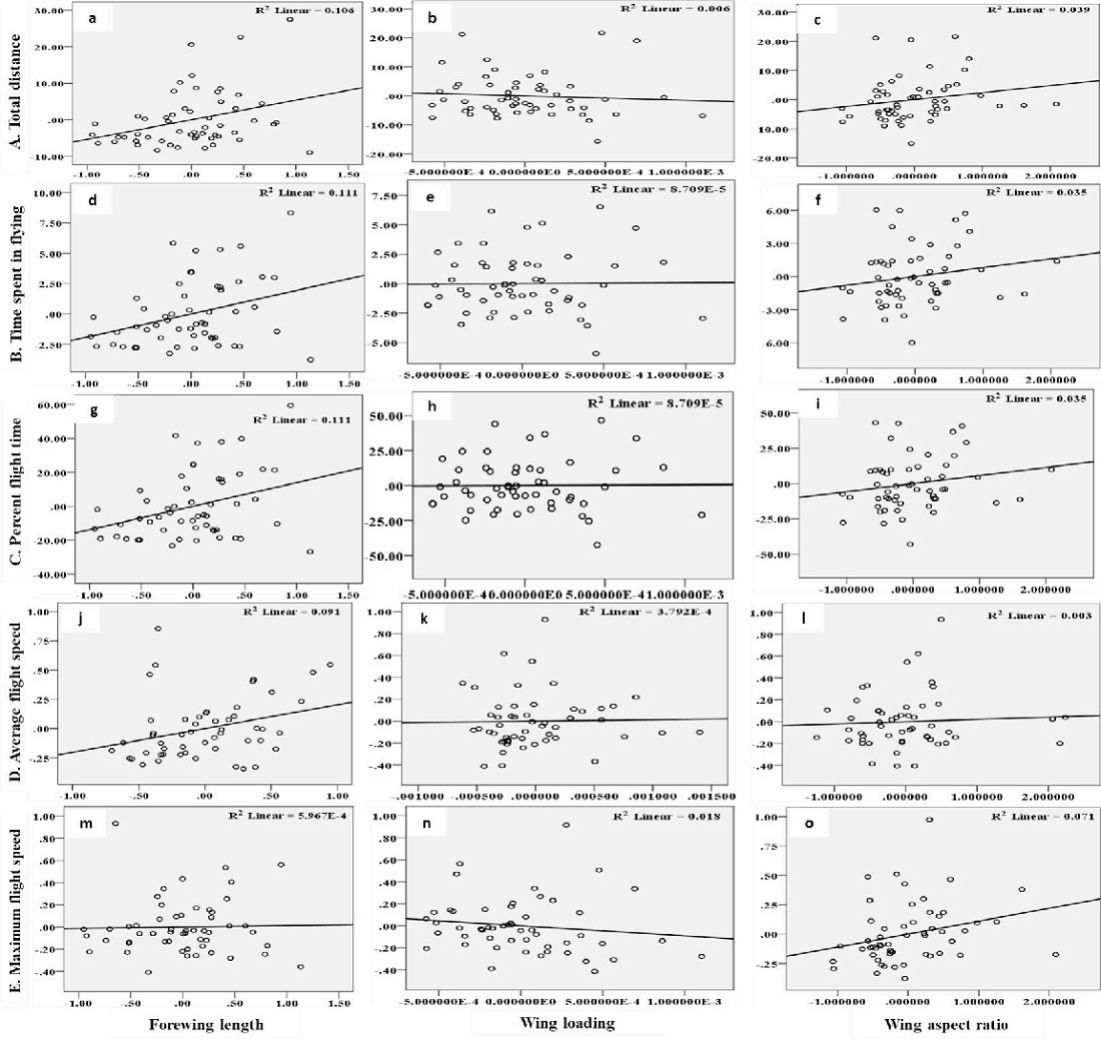
Graphs showing relationship between flight variables (dependent) with wing morphometrics (independent) in female moths of *H*. *armigera*. A) Total distance (a-c) B) Time spent in flying (d-f) C) Percent flight time (g-i) D) Average flight speed (j-l) and E) Maximum flight speed (m-o) as influenced by Forewing length, Wing loading and Wing aspect ratio.

In female moths, total distance travelled recorded positive significant relation with FWL but negative nonsignificant relation with WL and positive relation with WAR (Eq (2a); Fig 5a–5c). Females with greater FWL, WL and WAR engaged in flight for longer time (Eq (2b); Fig 5d–5f). Similar trend was noticed with respect to percent flight time (Eq (2c); Fig 5g–5i). However, average flight speed (Eq (2d); Fig 5j–5l) and maximum flight speed (Eq (2e); Fig 5m–5o) failed to establish any relation with FWL, WL and WAR.

$${\text{Y}}_{\text{TD}}=\;-73.518+5.370*{\text{X}}_{1}-1520.153{\text{X}}_{2}+2.384{\text{X}}_{3}+\varepsilon_{0}$$ (2a)

$${\text{Y}}_{\text{TSF}}=\;-27.967+1.942*{\text{X}}_{1\;}+65.650{\text{X}}_{2}+0.800{\text{X}}_{3}+\varepsilon_{0}$$ (2b)

$${\text{Y}}_{\text{PFT}}=-199.768+13.871*{\text{X}}_{1}+468.925{\text{X}}_{2}+5.714{\text{X}}_{3}+\varepsilon_{0}$$ (2c)

$${\text{Y}}_{\text{AFS}}=\;-0.731+0.013{\text{X}}_{1}-91.984{\text{X}}_{2}+0.110{\text{X}}_{3}+\varepsilon_{0}$$ (2d)

$${\text{Y}}_{\text{MFS}}=\;1.939-0.014{\text{X}}_{1}-483.474*{\text{X}}_{2}+0.151{\text{X}}_{3}+\varepsilon_{0}$$ (2e)

Where,

X_1_- Forewing length

X_2_- Wing loading

X_3_- Wing aspect ratio

## Discussion

Ability of long distance movement is one of the reasons for the successful establishment of *H*, *armigera* in vast crop ecosystems in Old world through obligatory and facultative migration [16, 45, 46]. The recent occurrence of this species in new world as well like in Brazil [47], Argentina [48], Bolivia [49], Paraguay and Uruguay [50] has justified its ability to move long distance. In India, *H*. *armigera* causes major economic loss in several agriculture/horticulture crops. Understanding movement trajectories of migrant populations, rate of dispersal, and their time and place of arrival helps formulate region-specific management strategies, assess biosecurity threats, develop region specific models and estimate spread of insecticide/*Bt* resistant populations

We used the flight-mill assay to evaluate migration activity in 1-day-old moths, based on earlier reports that flight behavior of *H*. *armigera* depends on age and mating [40]: 1–4 day old moths exhibited greater flight potential than older moths. Similarly, unmated females had better flight ability than mated moths [40, 51].

Both male and female *H*. *armigera* exhibited varied flight behavior. In the test population, 31 out of 106 moths performed very long-duration and long-distance flights, suggesting that *H*. *armigera* can migrate over great distances (as many as 40.92 km) in a single night. Moths were also capable of continuous flight bouts, flapping for nearly 68% of the flight time. Similar observations on long distance flight in heliothines have been observed across the globe (*H armigera*; [40, 50, 52]; *H*. *zea* [53], *Chloridea virescens* [54], *H*. *punctigera* [52]). However, not all moths exhibited long-range flights. Some (49) flew <5 km (mean = 1.61 km) with a maximum flight duration of 4 h (mean = 1.02 h), only 31% of their total test period. The remaining moths (26) were medium-range fliers (between 5 to 10 km, mean = 6.62 km) with a maximum flight duration of 6 h accounting for 33% of potential flight time. Globally, *H*. *armigera* exhibit both facultative and obligate migration, pointing to diverse strategies adopted by *H*. *armigera* to overcome ecological stress, including facultative diapauses [24], or surviving on alternate hosts [25].

When comparing the sexes, *H*. *armigera* males flew for a longer distance than females. In our study, the longest distance flown by a male was 40.92 km (with an overall average of 9.90 km), while the comparative figures for females were 33.76 km and 7.63 km. Mean maximum flight speed was greater (1.61 m/s) in females than males (1.53 m/s), but we observed no difference in average flight speed between sexes. This indicates that males engaged in slower, longer flights (typical of migration) more frequently than females. Male-biased flight performance has been previously observed in *H*. *armigera* [52; see also 55] and *H*. *punctigera*. *H*. *armigera* shows continuous variation in flight performance with individuals flying up to 40 km in a single night [27]. Similar results were obtained in *Agrotis ipsilon* (Hufnagel) [56], *Mythimna unipuncta* (Haworth) [37] and *Spodoptera* species (*S*. *litura* (F.) and *S*. *exigua* (Hübner)) [51]. This may be because females due to their ovaries are generally heavier than males, who carry a lighter payload [37, 57].

To study if morphological variation relates to migratory flight, we measured flight performance relative to morphology in *H*. *armigera* females and males. Our data show correlation between body morphometrics and flight, as also observed in other Lepidoptera [32, 58–61]. Wing length is an important correlate of flight speed and dispersal in butterflies [58, 59, 60]. In the present study forewing length was established as the most influencing parameter for long distance movement and grater flight ability. The wing loading was the next best wing parameter especially for male moths. In Lepidoptera, greater wing loading may allow longer and faster flights, and lower wing loadings are associated with slower flights and hovering [62, 63]. Migrant Lepidoptera and birds tend to have greater wing aspect ratios [64–67], indicating that high aspect ratios may impart greater dispersal ability. However, in the present study wing aspect ration failed to establish any significant relation with the flight parameters.

Together, these results show that *H*. *armigera* populations in South India can migrate, and that there are sex-specific and morphometry-based differences in their migration ability.

## Conclusions

South Indian population of *H*. *armigera* exhibited facultative migratory behavior in the present experimental studies, which is the first record from India. Both the sexes are equally capable of flying for longer duration covering greater distance, though, males overtake females in these tasks. The presented data provides ample evidence in the form of migrating individuals possessing greater forewing length, higher wing loading and wing aspect ratio over non-migratory individuals. Understanding the migratory behavior in economically important crop pests such as this would greatly help in tracking the movement trajectories of individuals which not only throw light on their gene flow but also help in developing suitable, ecofriendly management strategies.

## Supporting information

S1 File (PDF)
