# Supplementary material for: Evidence for facultative migratory flight behavior in Helicoverpa armigera (Noctuidae: Lepidoptera) in India
Source: PLoS One. 2021 Jan 22;16(1):e0245665. doi: 10.1371/journal.pone.0245665 (PMC7822321; doi:10.1371/journal.pone.0245665)
Supplement: S1 File — (PDF) [file pone.0245665.s001.pdf]

To derive tethered flight variables extracted from flight mill data through principal components analysis

In this study, basic flight data (distance flown every five seconds) a from flight mill under laboratory conditions from which 16 different variables (Table 1) were derived and assessed for levels of covariation among them using principal components analysis (PCA). In order to maximise the number of sample size for PCA both female and male data were combined.

The result of PCA gave the *Eigenvalues* corresponding to each principal component representing the variance associated with the particular principal component. The first *Principal component* (7.16) recorded maximum variation (44.76 per cent). The second *Principal component* (2.77) indicated second highest variability (17.31 per cent) followed by third *Principal component* (1.86) with third highest variability (11.61per cent) and so on up to 16 components. However, the first five Principal components which explain a total of 87.62 per cent variation in the present data were considered (Table 2).

The first five components score was taken as independent variables whereas; the *total distance* was taken as a dependent variable and the data was analysed by regression. The results of principal components regression indicated that first (PC1), second (PC2), third (PC3) and fifth (PC5) principal components were significant and have a positive influence on the *total distance* (F=135.24; p=0.01). Since the fourth (PC4) principal component has a negative influence on the *totaldistance* and was non-significant, it was not considered. The computed R^2 value (0.87) indicated that around 87 per cent of the variations in dependent variable was explained by these five principal components (Table 3).

Based on *rotated component matrix*, the variables which exhibited the factor loading of 0.8 and above were selected from PC1, PC2, PC3 and PC5. Accordingly, longest flight duration with factor loading of 0.899, furthest flight distance (0.892),longest flight distance (0.891), total duration (0.856), total distance (0.833),average flight duration (0.819), average flight distance (0.809) under PC1;longest flight average speed (0.881) under PC2;first flight duration (0.949) andfirst flight distance (0.936) under PC3 andnumber of flights (0.874) under PC5 were selected. None of the variables under PC4 could be considered as they were non-significant (Table 4).

S1 Table: Measured and derived tethered flight variables extracted from flight mill data.

Tethered flight variable	Definition	Units
**Total distance**	Sum of distance covered by all flights	metres
**Total duration**	Sum of duration of all flights	seconds
**Number of flights**	Count of flights	numeric
**Average flight distance**	Mean of distances of flights	metres
**Average flight duration**	Mean of duration of flights	seconds
**Average flight speed**	Mean of the speeds of individual flights (calculated as distance/duration)	metres/sec
**Maximum speed attained**	Greatest distance attained in any 5 second interval/5 – of the whole night	metres/sec
**First flight distance**	Distance of first flight of the night	metres
**First flight duration**	Duration of first flight of the night	seconds
**First flight average speed**	Speed of first flight of the night(calculated as distance/duration)	metres/sec
**First flight max speed**	Greatest speed attained in any 5 second interval of the first valid flight	metres/sec
**Furthest flight distance**	Distance travelled in the flight of greatest distance of the whole night	metres
**Longest flight distance**	Distance travelled in the flight of greatest duration of the whole night	metres
**Longest flight duration**	Duration of the flight with greatest duration	seconds
**Longest flight average speed**	Speed of the flight with greatest duration(calculated as distance/duration)	metres/sec
**Longest flight max speed**	Greatest speed attained in any 5 second interval of the flight of greatest duration	metres/sec

S2 Table: Eigenvalues with total variance among flight variables in *H. armigera* obtainedthrough Principal components analysis (PCA)

Components	Eigenvalues	Variance (%)	Cumulative (%)
**PC1**	7.16	44.76	44.76
**PC2**	2.77	17.31	62.08
**PC3**	1.86	11.61	73.69
**PC4**	1.23	7.66	81.35
**PC5**	1.00	6.27	87.62
**PC6**	0.61	3.80	91.42
**PC7**	0.55	3.40	94.82
**PC8**	0.34	2.10	96.92
**PC9**	0.18	1.15	98.07
**PC10**	0.14	0.86	98.93
**PC11**	0.09	0.53	99.47
**PC12**	0.06	0.35	99.82
**PC13**	0.02	0.10	99.91
**PC14**	0.01	0.06	99.97
**PC15**	0.00	0.03	100.00
**PC16**	0.00	0.00	100.00

S3 Table: Regression analysis for the Principal component loadings

	**Intercept**	**PC1**	**PC2**	**PC3**	**PC4**	**PC5**
**Coefficients**	8746.695**	8517.855**	3776.393**	1019.917**	-22.181	1778.293**
**t- statistic value**	23.95**	23.22**	10.29**	2.78**	-0.06	4.85**
**F- value**	135.24**					
**R^2 value**	0.87					

** Significant @ 0.01

S4 Table: Rotated component matrix for selection of flight variables in *H. armigera* through Principal Component (PC) loadings

Flight variables	PC1	PC 2	PC 3	PC 4	PC 5
**Longest flight duration**	0.899*	0.064	0.277	0.058	-0.03
**Furthest flight distance**	0.892*	0.167	0.331	0.056	-0.079
**Longest flight distance**	0.891*	0.168	0.329	0.056	-0.079
**Total duration**	0.856*	0.234	0.102	-0.03	0.325
**Total distance**	0.833*	0.369	0.1	-0.002	0.174
**Average flight duration**	0.819*	0.001	-0.107	0.307	-0.328
**Average flight distance**	0.809*	0.036	-0.115	0.299	-0.344
**Longest flight average speed**	-0.038	0.881*	0.062	0.186	-0.075
**Longest flight maximum speed**	0.249	0.79	0.181	0.194	0.109
**Average flight speed**	0.211	0.789	-0.19	0.155	-0.23
**Maximum speed attained**	0.307	0.716	0.07	0.254	0.328
**First flight duration**	0.204	0.01	0.949*	0.098	-0.043
**First flight distance**	0.207	0.06	0.936*	0.146	-0.043
**First flight average speed**	0.075	0.405	0.094	0.859	0.096
**First flight maximum speed**	0.211	0.327	0.242	0.827	0.141
**Number of flights**	-0.111	-0.021	-0.106	0.166	0.874*

***** The loadings of the variables which contribute maximum to the principal components (PC)

Relationship between flight variables and adult morphometrics of *Helicoverpa armigera*

Flight variables extracted from PCA viz.,*total distance, total duration, longest flight duration, furthest flight distance, longest flight distance, average flight duration, average flight distance, longest flight average speed, first flight duration, first flight distance* and *number of flights* were subjected to multiple correlation and stepwise regression with adult morphometry of *H. armigera* viz., forewing length, forewing area, forewing width, wing loading, wing aspect ratio and front wing quotient to know the relationship between flight variables and adult morphometrics and identify the most influencing parameters. The results pertaining to the same are presented hereunder.

Both male and female moths

Stepwise regression was adopted to identify the most influencing morphometric variable(s) for different tethered flight variables. Results indicated that forewing length had a significant positive influence on total distance (R^2= 0.16, F=19.32**), whereas, both forewing length and wing loading had a significant and positive influence on total duration (R^2= 0.20, F=12.70**). However, forewing length showed no influence on the longest flight average speed (R^2= 0.05, F=5.28*). Wing aspect had no influence on furthest flight distance (R^2= 0.05, F=5.04*) and longest flight distance (R^2= 0.05, F=5.04*). Similarly, wing loading exhibited no relation with longest flight duration (R^2= 0.04, F=4.25*) followed by first flight distance (R^2= 0.07, F=8.02*) and first flight duration (R^2= 0.08, F=8.91*), whereas, forewing width recorded significant positive influence on number of flights (R^2=0.07, F=7.41**). However, average flight distance and average flight duration did not show any relation with any of the adult morphometric parameters (Table 5).

S5 Table: Quantification of flight variables based on adult morphometrics of *Helicoverpa armigera* (Both male and female)

Flight variables (Response)	Adult morphometrics (Predictors)	Coefficients	F-value	t-value	R^2
**Total distance**	Constant	-80285.87	19.32**	-3.96**	0.16
	Forewing length	8338.15		4.40**	
**Total duration**	Constant	-128154.55	12.70**	-4.54**	0.20
	Forewing length	10363.68		4.43**	
	Wing loading	7.09E6		2.81**	
**Furthest flight distance**	Constant	-19237.24	5.04*	-1.89**	0.05
	Aspect ratio	1793.96		2.24**	
**Longest flight distance**	Constant	-19259.29	5.04*	-1.89**	0.05
	Aspect ratio	1795.14		2.25**	
**Longest flight average speed**	Constant	-1.16	5.28*	-1.43^{NS}	0.05
	Forewing length	0.17		2.30*	
**Longest flight duration**	Constant	-10100.45	4.25*	-1.37^{NS}	0.04
	Wing loading	3.48E6		2.06*	
**Average flight distance**	NS				-
**Average flight duration**					
**First flight distance**	Constant	-6773.70	8.02**	-2.50**	0.07
	Wing loading	1.75E6		2.83**	
**First flight duration**	Constant	-8031.03	8.91**	-2.63**	0.08
	Wing loading	2.08E6		2.99**	
**Number of flight**	Constant	-214.32	7.41**	-2.20*	0.07
	Forewing width	49.80		2.72**	

** Significant @ 0.01, * Significant @ 0.05, NS=Non-significant, R^2= Co-efficient of determination.
